# Identification of novel biomarkers related to pathogenesis and treatment of psoriasis based on integrated analysis of weighted gene co-expression network analysis and LASSO

**DOI:** 10.1371/journal.pone.0317666

**Published:** 2025-06-25

**Authors:** Chenguang Wang, Zhiyong Liu, Yan He, Yashu Zhang, Shiqi Chen, Yuhao Zhou, Wenqing Yang, Lijun Fan

**Affiliations:** 1 Centre for Endemic Disease Control, Chinese Centre for Disease Control and Prevention, Harbin Medical University, Harbin, China; Kwame Nkrumah University of Science and Technology, GHANA

## Abstract

**Background:**

Psoriasis is an inflammatory skin disease, and current treatments have their own limitations, including moderate treatment effectiveness, poor compliance, and potential safety risks, etc. Therefore, the primary focus of this study is to explore novel molecular targets and improve the diagnosis and treatment of psoriasis patients.

**Method:**

In this study, comprehensive bioinformatics analysis was performed on the expression profiles of tissue samples from patients with psoriasis in the clinical trial of TYK2/JAK1 inhibitor treatment (NCT02310750). Weighted gene co-expression network analysis (WGCNA) and least absolute shrinkage and selection operator (LASSO) regression were performed to identify characteristic genes and construct the diagnostic models. Gene set enrichment analysis (GSEA) was used to identify the biological processes of psoriasis characteristic gene sets. GO and KEGG pathway analysis were combined to elucidate the potential biological significance of differentially expressed genes (DEGs). The accuracy of biomarker identification was further validated using immune cell infiltration and receiver operating characteristic (ROC) curves based on external data (GSE6710\GSE30999\GSE14905).

**Results:**

A total of 5 genes (*DEFB103A, OAS3, OASL, SAMD9, STAT1*) were co-identified as characteristic genes in psoriasis progression and treatment. The feature of the immune cell infiltration was highly consistent with association of characteristic biomarkers with immune cells. A total of 14 up-regulated genes and 5 down-regulated genes were identified in respective modules (AUC _NL/LS_ = 0.9783; AUC _pre/post_ = 0.9395; AUC _external_ = 0.9469). In addition, 8 genes (*DEFB103A, OASL, HERC6, ISG15, MKI67, MX1, MXD1, SCO2*) were considered to have statistically significant differences in sensitivity of short-term treatment for psoriasis.

**Conclusion:**

The research findings provide an understanding of the role of novel biomarkers and offer a perspective for further in-depth investigation into the progression and treatment of psoriasis.

## 1. Introduction

Psoriasis is a chronic, recurrent, inflammatory and systemic disease caused by a combination of genetic predisposition and environmental factors. This immune-mediated disease clinically presents as plaques or patches of psoriasis, characterized by local or widespread distribution [1,2]. Psoriasis is difficult to treat and has a significant impact on the well-being of millions of adults and children worldwide. Epidemiological data revealed that psoriasis prevailed in approximately 19% of countries globally, with an uneven geographical distribution [3]. The quality of life of the majority of psoriasis patients is compromised to some extent, with many experiencing serious negative effects on their social and psychological well-being [4]. Psoriasis presents with a variety of clinical symptoms, chronic plaque psoriasis and the common form being the two most prevalent types. The typical morphological features are well-defined erythema patches covered with silvery scales on light skin and gray patches on dark skin [5]. In severe cases, it can progress to erythrodermic psoriasis, a severe and potentially life-threatening variant of the disease. The manifestations often include confluent erythema, scaling, or desquamation, affecting more than 75% of the body surface skin. The extent of skin involvement may be associated with factors such as low temperatures, high-output congestive heart failure, electrolyte imbalances, pruritus, and skin pain [6–8].

The pathogenesis of psoriasis is considered to be the consequence of dysregulated proliferation of keratinocytes, involving immune system dysregulation. The various phenotypic manifestations of psoriasis are mediated by the interplay of cytokines released by dendritic cells, T-helper1 (Th1), T-helper2 (Th2), and T-helper17 (Th17) cells. In the realm of immunology and genetics research on psoriasis over the past few decades, the identification and emphasis on the adaptive immune pathway involving Interleukin-17 (IL-17) and Interleukin-23 (IL-23) have emerged as potential factors [9,10]. The main mechanism involves the interaction between the innate and adaptive immune systems and the feed-forward amplification of psoriasis inflammatory factors [11]. Additionally, other factors such as tumor necrosis factor (TNFα), type I interferons, and antiviral signaling are involved in the pathogenesis. In recent decades, multiple pathways have emerged in research, among which the Janus kinase (JAK)/signal transducer and activation of transcription (STAT) pathway has been recognized and extensively studied as a critical signaling pathway. JAK, a non-receptor tyrosine kinase, is involved in the transduction of cytokine signaling [12,13]. Upon activation and phosphorylation, JAK forms a dimer and subsequently activates STAT. Activated STAT can dimerize and translocate to the nucleus to regulate transcription, thereby further modulating the inflammatory mechanisms underlying psoriasis. STAT1 conducts type I interferon and type II interferon signaling, which allows Interferon-γ (IFN-γ) to sensitize keratinocytes and allow inflammatory cells to enter the psoriasis lesion stage. STAT3 plays a pivotal role in promoting the induction and differentiation of Th17 cells. Furthermore, STAT3 can be activated through the IL-23-induced JAK2/TYK2 (Tyrosine Kinase 2) pathway and also through the IL-6-induced JAK1/JAK2 and JAK1/TYK2 pathways [14–16]. Consequently, it leads to a further amplification of psoriasis inflammation through a positive feedback mechanism.

The current main treatment modalities include emollients, phototherapy, corticosteroids, vitamin D analogs, calcineurin inhibitors, keratolytics, oral systemic therapies, and biologic agents [17,18]. However, the current various treatment modalities have limitations. Topical medications have poor compliance and relatively low efficacy [19]. Phototherapy exhibits high selectivity for certain subtypes of psoriasis and can cause skin damage [17]. Methotrexate carries safety risks primarily related to bone marrow suppression and gastrointestinal complication [20]. Cyclosporine poses irreversible nephrotoxicity risks [21]. Although dimethyl fumarate has been approved by the European Medicines Agency, it can still cause flushing and diarrhea with increasing doses [22]. Four categories of biologic agents (anti-TNFα, anti-IL-17, anti-IL-12p40 or IL-23p40, and anti-IL-23p19) have distinct treatment mechanisms and safety profiles [23,24]. However, the highly specific targeting of inflammatory mediators by biological agents may result in immune responses that bypass blockade, leading to exacerbation and associated changes in clinical and immunological characteristics [25–27].

Weighted Gene Co-expression Network Analysis (WGCNA) is a systematic biological method that is employed to elucidate the patterns of interrelatedness among genes within microarray samples. WGCNA is specifically used to identify clusters or modules of highly correlated genes, and these clusters are summarized by the feature genes within the module [28]. Emphasis is placed on identifying hub genes that exhibit a strong association with clinical features, thereby qualifying them as highly relevant biomarkers and therapeutic targets in relation to the respective diseases.

GSE136757, the data set from a clinical trial (NCT02310750, Study Registration Date was 2014-11-18) involving patients with moderate to severe psoriasis in GEO database (Gene Expression Omnibus) [29]. By examining the expression profiling characteristics of non-lesional (NL)/lesional (LS) skin before treatment of clinical trial drug TYK2/JAK inhibitor (PF-06700841) and lesional skin that pre/post treatment in patients with psoriasis, an integrated approach combining analysis of differentially expressed genes (DEGs) and WGCNA based on clinical features was employed. To identify the co-expressed gene sets related to the pathogenesis, treatment and short-term treatment sensitivity of psoriasis, and perform related functional enrichment to analyze the characteristics and functions of genes.

## 2. Materials and methods

### 2.1. Data source

In this research, the expression profile dataset of patients with moderate and severe psoriasis before and after treatment (GSE136757) was obtained from GEO public database. GSE136757 contained transcriptome data of moderate to severe psoriasis patients who were randomly assigned (30 mg PF-06700841, 100 mg PF-06700841or placebo) for continuous treatment for 28 days. Expression profiles of 26, 56 and 162 NL/LS samples in GSE6710, GSE14905 and GSE30999 datasets were selected as external datasets to validate the key genes in this study. The data from the GEO database is free of charge, and using this data does not require approval from an Ethics Committee. The author ensure that all procedures were performed in compliance with relevant laws and institutional guideline.

### 2.2. Gene set enrichment analysis (GSEA)

GSEA algorithm was used to analyze psoriasis related biological process (BP), cellular component (CC), molecular function (MF) and Kyoto Encyclopaedia of Genes and Genomes (KEGG) pathways. The GSEA reference datasets are c5.go.bp.v2023.1.Hs.symbols.gmt, c5.go.cc.v2023.1.Hs.symbols.gmt, c5.go.mf.v2023.1.Hs.symbols.gmt, c2.cp.kegg.v2023.1.Hs.symbols.gmt in the MSigDB V2023.1.Hs database [30].

### 2.3. Identification of DEGs

The original dataset GSE136757 was normalized by log base 2 transformation. GPL-55999 dataset from the GPL570 platform was used to annotate and remove duplicates from the gene IDs in the original GSE136757 dataset. “Limma” package in R software version 4.3.0 was used to identify DEGs between the lesional and non-lesional skin of psoriasis patients before administration, as well as the lesional skin pre/post treatment. Genes with a p-value less than 0.05 and |log2 fold change| greater than 0.5 were considered as DEGs.

### 2.4. Construction of WGCNA network

WGCNA, which could describe the patterns of correlation between genes in microarray samples, was used to identify modules of highly correlated genes. These modules can be summarized using module characteristic genes or hub genes, and also can be associated with clinical features using the feature gene network method, resulting in gene module membership and statistical significance. The “WGCNA” package in R version 4.3.0 was used to further identify the networks relationship of DEGs.

In order to ensure that the network is scale-free, the soft threshold power for the construction of gene co-expression networks in NL/LS was 12, and pre/post treatment was 15. Based on the parameters (deepSplit = 2, minModuleSize = 30, mergeCutHeight = 0.3), the genes with similar expression profiles were divided into different modules using dynamic tree-cutting method. To more fully understand the co-expression relationships between genes and reveal more meaningful biological insights, WGCNA analysis results were also supplemented with raw expression data from the entire transcriptome (soft threshold power = 13 in NL/LS, pre/post treatment was 11; deepSplit = 2, minModuleSize = 100, mergeCutHeight = 0.3). The topological overlap matrix (TOM) was established to measure the average network connectivity of each gene. To refine the selection of module eigengenes (MEs), a hierarchical clustering approach was employed to construct a dendrogram, which was used to calculate the correlation between MEs and clinical phenotypes (NL/LS, pre/post treatment). The module that showed the highest correlation with psoriasis onset or treatment outcomes among all modules was determined to be the most crucial module for further analysis. The selection criteria for hub genes in the key modules of the co-expression network of expression profiles in NL/LS cohort were gene significance (GS) greater than 0.9 and a module membership (MM) greater than 0.9 (pre/post treatment cohort, GS > 0.8, MM > 0.9).

### 2.5. Functional enrichment analysis

In order to further clarify the biological significance of key modules in the occurrence, progression, and treatment of psoriasis, GO (Gene Ontology) and KEGG (Kyoto Encyclopedia of Genes and Genomes) pathway analysis were performed within the key modules [31–33]. The “clusterProfiler” package in the R software was used for elucidating biological process (BP), molecular functions (MF), and cellular components (CC), and KEGG pathways. GO or KEGG pathways with an FDR < 0.01 were considered significant. Dot plot and target network diagram were constructed to visualize GO and KEGG analysis results. Target network relationship diagrams were created using Cytoscape, which provided a comprehensive view of the interactions between the different genes and pathways involved in the analysis.

### 2.6. Immune cell infiltration

CIBERSORT, a method for deconvolution of the expression matrix of 22 human immune cell subtypes using linear support vector regression principles for the difference of immune cells between skin lesions and non-skin lesions in patients with psoriasis [34]. Subsequently, the correlation between characteristic genes and immune cells was analyzed by spearman method to further confirm the role of characteristic genes in psoriasis.

### 2.7. LASSO model and ROC curve

LASSO (least absolute shrinkage and selection operator) is a method for building generalized linear models and selecting variables. It belongs to the class of shrinkage estimators, where some of the regression coefficients are directly shrunk to zero during the process of coefficient reduction, thereby achieving the function of variable selection. This workflow involves the “glmnet” package in R software version 4.3.0 [35,36].

In addition, Receiver operating characteristic (ROC) curves were used to verify the stability and sensitivity of the LASSO model for genetic screening of NL/LS and pre/post treatment of skin lesions [37]. In order to ensure the stability of verification results, the genes identified by LASSO were verified based on an external data set GSE30999. The analysis was performed using the “pROC” package in R software version 4.3.0.

## 3. Results

### 3.1. Identification of DEGs

**Fig 1:**
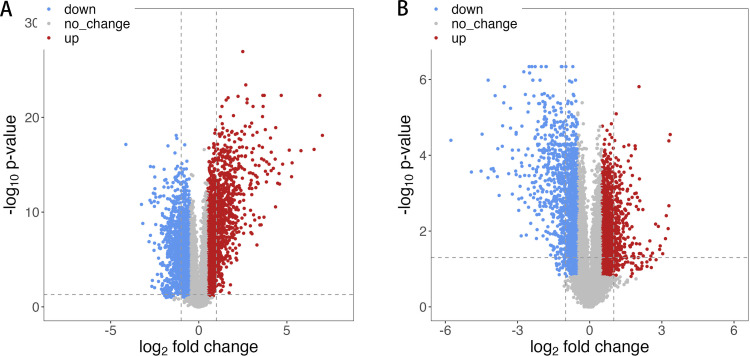
Visualization plots of differentially expressed genes. **Up-regulated genes (red dots) and down-regulated (blue dots).** (A)Volcano plot in non-lesional (NL)/ lesional (LS); (B) Volcano plot in pre/post treatment.

In GSE136757, DEGs analysis was performed on the expression profiles of NL/LS before treatment and the lesional skin of patients with favorable treatment outcomes (patients were treated continuously for 14 or 28 days and had post treatment gene expression profiles in the lesions). In order to recognize the genes significantly correlated with the occurrence, progression, and treatment of psoriasis, we identified the DEGs in non-lesional/lesional before treatment, as well as the DEGs that showed significant changes pre/post treatment in the psoriatic lesional skin. A total of 1901 upregulated genes and 2136 downregulated genes were found as DEGs in the NL/LS cohort (S1 Table). In the pre/post treatment cohort, 1499 upregulated genes and 1559 downregulated genes were found (S2 Table). DEGs were presented with volcano plots (Fig 1), and the top 50 DEGs were shown by heatmaps in S1 Fig.

### 3.2. Functional enrichment analysis

**Fig 2:**
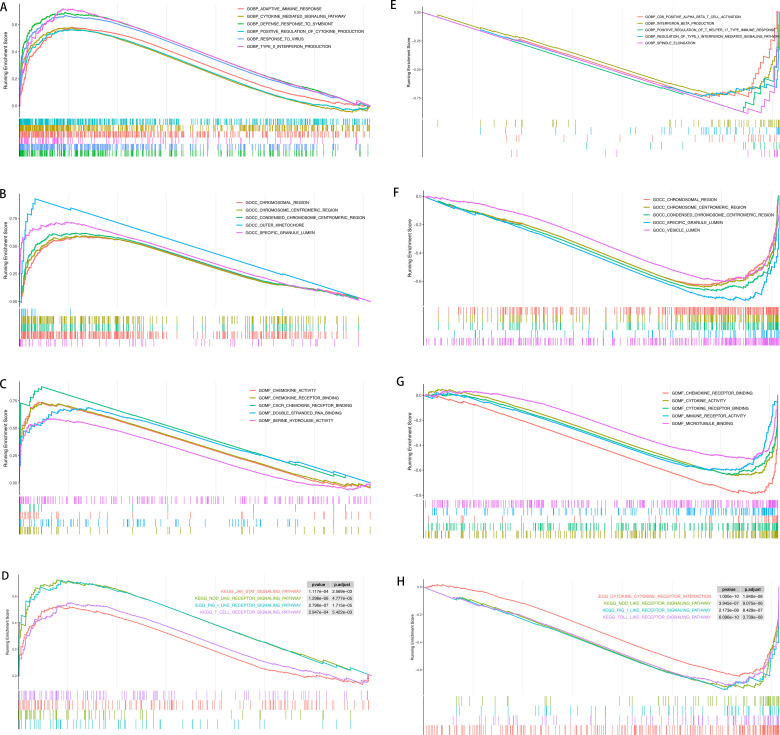
Gene set enrichment analysis. **NL/LS cohort.** (A) Biological process; (B) Cellular component; (C) Molecular function (D) KEGG pathway; Pre/Post treatment cohort: (E) Biological process; (F) Cellular component; (G) Molecular function; (H) KEGG pathway.

GSEA was performed for DEGs in NL/LS and pre/post treatment, respectively. Multiple genomes of the BP term type such as adaptive immune response, cytokine-mediated signaling pathway, and type II interferon production were significantly enriched in diseased skin compared to controls (Fig 2A). Simultaneously, a total of 35 CC term gene sets, including chromosomal region, condensed chromosome centromeric region, and outer kinetochore, were significantly enriched (Fig 2B). Compared with the control group, the expression profile of the lesional skin exhibited a significant enrichment in MF terms such as chemokine activity, chemokine receptor and double-stranded RNA binding (Fig 2C). Notably, MF terms closely resemble the enrichment types observed in BP. Through further investigation of the KEGG pathway, it was observed that the group of skin lesions exhibited significant enrichment in the JAK-STAT signal pathway, NOD-like receptor pathway, RIG-I-like receptor pathway, and T cell receptor pathway (Fig 2D).

The GSEA results for DEGs expression profiles of pre/post treatment groups in psoriasis are presented in Fig 2E–H. According to the GO analysis of BP shown in Fig 2E, a notable enrichment of BP terms associated with various psoriasis biological processes, including CD8 + alpha-beta T cell activation, interferon β production and T helper 17 type immune response, etc. The results were mainly enriched in 62 CC term sets, including specific granule lumen, vesicle lumen, condensed chromosome and centromeric region, etc. These results were similar to the GSEA results of the lesional and non-lesional cohort (Fig 2F). In the MF class, such as chemokine receptor binding, cytokine activity, and immune receptor activity were significantly enriched compared to controls (Fig 2G). KEGG pathway results showed that significant enrichment in pathways such as Cytokine-cytokine receptor interaction, NOD-like receptor, RIG-I-like receptor, and Toll-like receptor pathway (Fig 2H).

### 3.3. Identification of hub genes within key modules based on WGCNA

**Fig 3:**
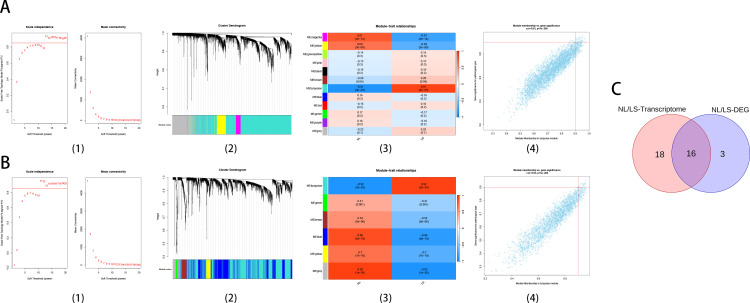
Weighted gene co-expression network analysis in NL/LS cohort. (A) Whole Transcriptome, (B) Differentially expressed gene. (1) Analysis of the scale-free fit index for various soft-thresholding powers and analysis of the mean connectivity for various soft-thresholding powers, (2) Clustering dendrogram, (3) Module-trait associations evaluated by correlations between MEs and clinical traits, Red is positively correlated with clinical traits, and blue is negatively correlated with clinical traits, (4) The correlation of module membership and gene significance in the turquoise module. (C): Venn plot showing the overlapping genes.

Whole Transcriptome data of 60 NL/LS samples from the GSE136757 data were used for WGCNA analysis. When the soft threshold power was confirmed as 12, the scale independence (scale-free topological model fitting, signed R^2^) reached 0.85, and the adjacency matrix obtains a higher average connectivity value (Fig 3A). A total of 12 distinct modules were identified and analyzed based on the dynamic tree. These modules were analyzed together with clinical phenotypes to obtain a network of module-trait relationships associated with clinical outcomes, and the turquoise labeled modules with the highest association with psoriasis progression (r = 0.93, P = 3e^-23^). The correlation results of MM (module membership) and GS (gene significance) of each gene in turquoise module showed that the overall correlation of turquoise module reached 0.91 (P < 1e ^− 200^). The screening criteria of hub gene were set as GS > 0.9 and MM > 0.9, and 34 hub genes were identified as up-regulated for further analysis. A total of 4037 (2136 down-regulated genes, 1901 up-regulated genes) DEGs in 60 samples were screened in NL/LS cohort (S2 Fig). When the soft threshold power was confirmed as 12, the scale independence (signed R^2^) reached 0.85, and the adjacency matrix gained a comparatively higher mean connectivity value. Subsequently, 6 distinct modules obtained from dynamic tree cutting with the parameters minModuleSize = 30 and mergeCutHeight = 0.3. The network of module-trait relationships associated with clinical outcomes was shown in Fig 3G. The module highlighted in turquoise color exhibited the strongest correlation with the progression of psoriatic lesions (r = 0.92, P = 2e^-25^). The correlation between the MM (module membership) and GS (gene significance) of each gene in the turquoise module was analyzed. It was found that the overall correlation of the turquoise module reached 0.93 (P < 1e^−200^). In the screening criteria for GS > 0.9 and MM > 0.9, a total of 19 hub genes were identified with up-regulated expression in this module for further analysis (Fig 3B). Ultimately, we identified 16 intersection genes (ABCG4, CD24, DEFB103A, EPHB2, FABP5, HPSE, IFI16, KYNU, OAS3, OASL, PLA2G4D, PRSS27, SAMD9, SERPINB13, STAT1, TMPRSS11D) that have been designated as candidate hub genes that may be critical in the onset and progression of psoriasis (Fig 3C). Most of these genes (CD24, EPHB2, FABP5, HPSE, KYNU, OAS3, OASL, PRSS27, SAMD9, SERPINB13, STAT1, TMPRSS11D) were also identified in hub genes of the module most associated with psoriasis progression in external datasets GSE6710/GSE14905 (S3 Table).

**Fig 4:**
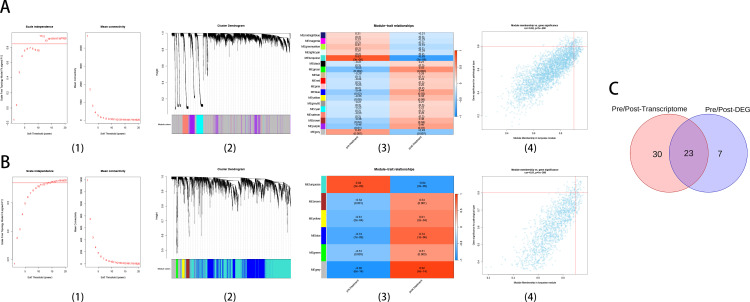
Weighted gene co-expression network analysis in pre/post treatment cohort. A: Whole Transcriptome, B: Differentially expressed gene. (1) Analysis of the scale-free fit index for various soft-thresholding powers and analysis of the mean connectivity for various soft-thresholding powers, (2) Clustering dendrogram, (3) Module-trait associations evaluated by correlations between MEs and clinical traits, Red is positively correlated with clinical traits, and blue is negatively correlated with clinical traits, (4) The correlation of module membership and gene significance in the turquoise module. (C): Venn plot showing the overlapping genes.

WGCNA analysis was also performed on all pre/post treatment mRNA expression profiles with the soft threshold to 11. Modules in turquoise showed the strongest association with psoriasis treatment outcomes in the modular trait map (r = 0.83, P = 3e^-8^). The overall correlation of genes in this module also reached 0.82 (P < 1e^-200^). A total of 53 hub genes were identified base on down-regulated expression in the screening criteria for GS > 0.8 and MM > 0.9 (Fig 4A). The sample clustering outlier detection of pre/post treatment cohort is shown in S3 Fig. In the construction of a weighted gene co-expression network for 3058 differentially expressed genes (1499 upregulated genes, 1559 downregulated genes) in pre/post treatment cohort of psoriasis patients in 36 samples. Scale-free topology model fit (signed R^2^) of 0.85 was set for scale independence with the soft threshold of 15. The turquoise module exhibited the strongest correlation with psoriasis treatment prognosis in the modular trait map (r = 0.84, P = 2e^-9^). The overall correlation of genes within turquoise module also reached 0.81 (P < 1e^-200^). In the screening criteria for GS > 0.8 and MM > 0.9, a total of 30 hub genes were identified with down-regulated expression in this module for further analysis of progression and treatment of psoriasis (Fig 4B). In pre/post treatment cohort, we identified 23 intersection genes (BUB1, CCNB1, CKS2, DEFB103A, HERC6, IFI16, ISG15, MKI67, MX1, MXD1, NCAPG, OAS2, OAS3, OASL, RSAD2, SAMD9, SCO2, STAT1, TRIM14, UBE2C, UBE2F, UBE2L6, UHRF1) that have been designated as candidate hub genes that may play a key role in the treatment of psoriasis (Fig 4C). More than half of the genes were also included in hub genes up-regulated in external datasets, even when the datasets did not involve psoriasis drug therapy. (S3 Table).

Notably, among the 16 and 23 hub genes identified in two cohorts, 5 genes (DEFB103A, OAS3, OASL, SAMD9, STAT1) were simultaneously identified, however, their expression levels were significantly opposite. OAS3, OASL, SAMD9 and STAT1 were all included in the hub genes identified by the external validation data (GSE6710/GSE14905). These results indicated that the expression levels of these co-identified biomarkers were correlated with both the pathogenesis of psoriasis and the clinical drug treatment mechanism (TYK2/JAK1 inhibitor).

### 3.4. GO and KEGG pathway analysis of key modules

**Fig 5:**
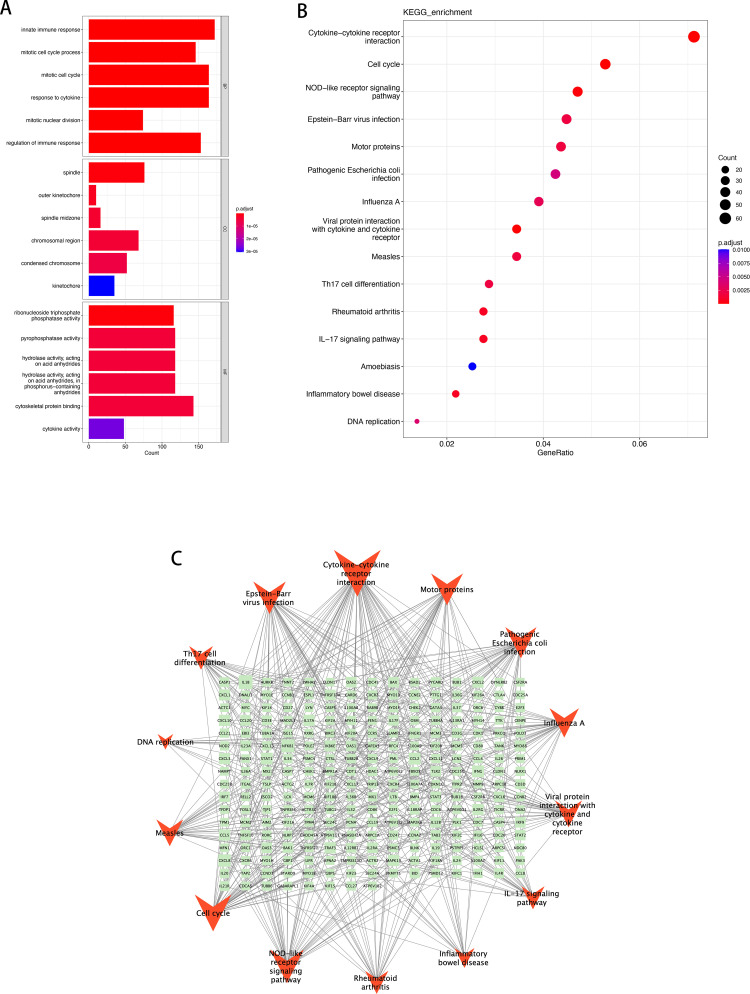
Functional enrichment analysis and interaction network of gene pathways of turquoise module in NL/LS cohort. (A) The top 6 functional enrichment in BP, CC, and MF analysis, respectively; (B) The results of KEGG analysis; (C) Topological analysis result was calculated using degree, the light green rectangles represent the corresponding genes, and the red angles represent different signal pathways. A larger size represents a larger degree.

The enrichment pathway analysis of key modules in the WGCNA analysis results of differentially expressed genes was performed with the condition of p.adjust < 0.01. 436 enriched GO-BP pathways, 35 GO-CC pathways, and 19 GO-MF pathways were identified in NL/LS cohort (S4 Table). The enriched GO-BP pathways were mainly associated with immune and cell cycle-related pathways. Regarding BP enrichment, the turquoise module primarily participated in innate immune response, mitotic cell cycle process, mitotic cell cycle, and so on. Apart from that, pathways related to inflammation and interferon response, such as inflammatory response and type II interferon, were also highly enriched. The GO-CC pathways were enriched with high levels of terms such as spindle, outer kinetochore, and chromosomal region. The remarkably enriched MF terms included ribonucleoside triphosphate phosphatase activity, pyrophosphatase activity, hydrolase activity, acting on acid anhydrides, etc. (Fig 5A, chord diagram in S4–S6 Figs). Furthermore, based on KEGG analysis, significant enrichment was observed in Cytokine-cytokine receptor interaction, IL-17 signaling pathway, Th17 cell differentiation, and NOD-like receptor signaling pathway, etc. Visualization and results are shown in Fig 5B and S5 Table.

**Fig 6:**
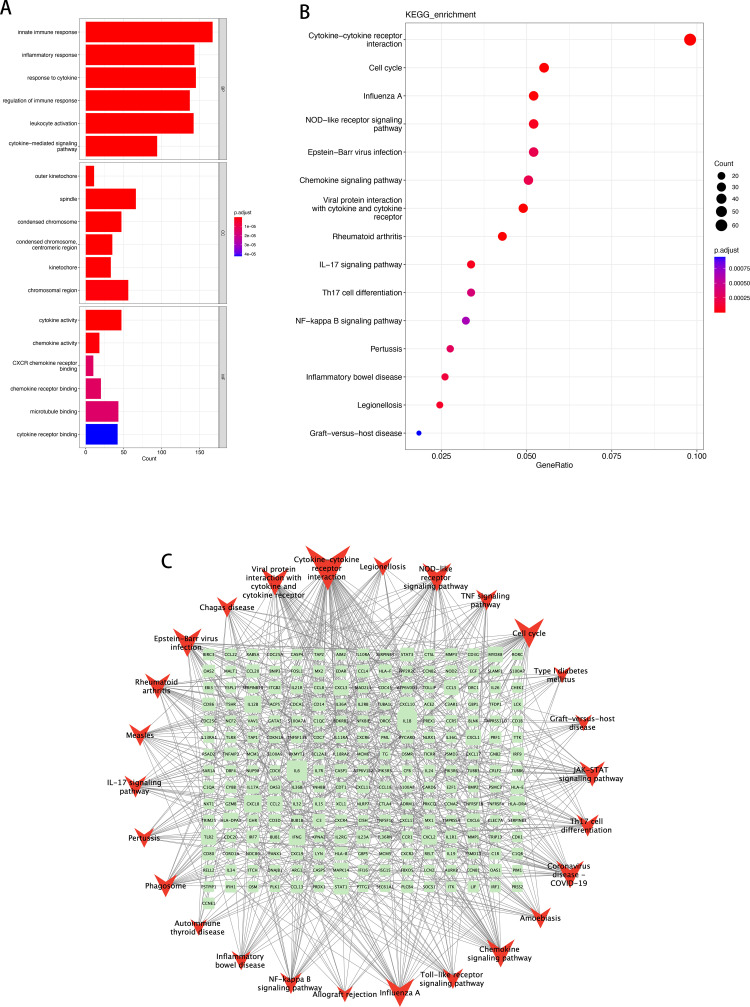
Functional enrichment analysis and interaction network of gene pathways of turquoise module in pre/post treatment cohort. (A) The top 6 functional enrichment in BP, CC, and MF analysis, respectively; (B) The results of KEGG analysis; (C) Topological analysis result was calculated using degree. The light green rectangles represent the corresponding genes, and the red angle Vs represent different signal pathways. A larger size represents a larger degree.

In the exploration of the biological functions of key modules in pre/post treatment, a total of 623 GO-BP pathways, 49 GO-CC pathways, and 31 GO-MF pathways were enriched (S6 Table). The results of the exploration of GO biological functions in the pre/post treatment cohort were highly similar to the enriched pathways in the NL/LS cohort. GO-BP enrichment included pathways related to immune, inflammatory, and cytokine, such as innate immune response, inflammatory response, and response to cytokine. GO-CC enrichment is observed in cell cycle terms, such as spindle and outer kinetochore. GO-MF was enriched with terms including cytokine activity and chemokine receptor binding (Fig 6A, chord diagram in S7–S9 Figs). The repeated enrichment in the GO pathways indicates that biological processes or molecular functions centered around immune response and cytokine interaction may serve as crucial pathways in the pathogenesis of psoriasis.

In addition to the high degree of similarity observed in the GO analysis, the KEGG pathway enrichment analysis results also showed significant similarity (Fig 6B, S7 Table). KEGG analysis results of both cohorts showed consistent enrichment of highly enriched pathways, suggesting that Th17 (T-helper 17) and IL-17 (Interleukin 17) as the mechanism core of cytokine interaction may be a crucial pathway for the progression and treatment of psoriasis. The NOD-like receptors signaling pathway, which regulates immune response and inflammation, may be involved in the onset and development of the disease. Additionally, the relationship between Epstein-Barr virus infection and psoriasis may be closely linked. Furthermore, the molecular pathway mechanisms of rheumatoid arthritis (RA), a systemic autoimmune disease, may share a high degree of similarity with psoriasis.

Furthermore, a gene pathway network was constructed using Cytoscape based on KEGG signaling pathways and corresponding enriched genes (Figs 5C and 6C). It reveals that a that interleukin-associated nodes have higher gene pathway associations, such as IL-12B, IL1B, IL6, CXCL8. As well as nodes related to interferons, such as IFNG. GO and KEGG enrichment analysis revealed a significant enrichment of activity in both Cytokines and Chemokines, and it strongly suggested that these aforementioned nodes play a pivotal role as crucial biological nodes or mechanisms involved in the pathological progression of psoriasis.

### 3.5. Immune cell infiltration in characteristic genes

**Fig. 7:**
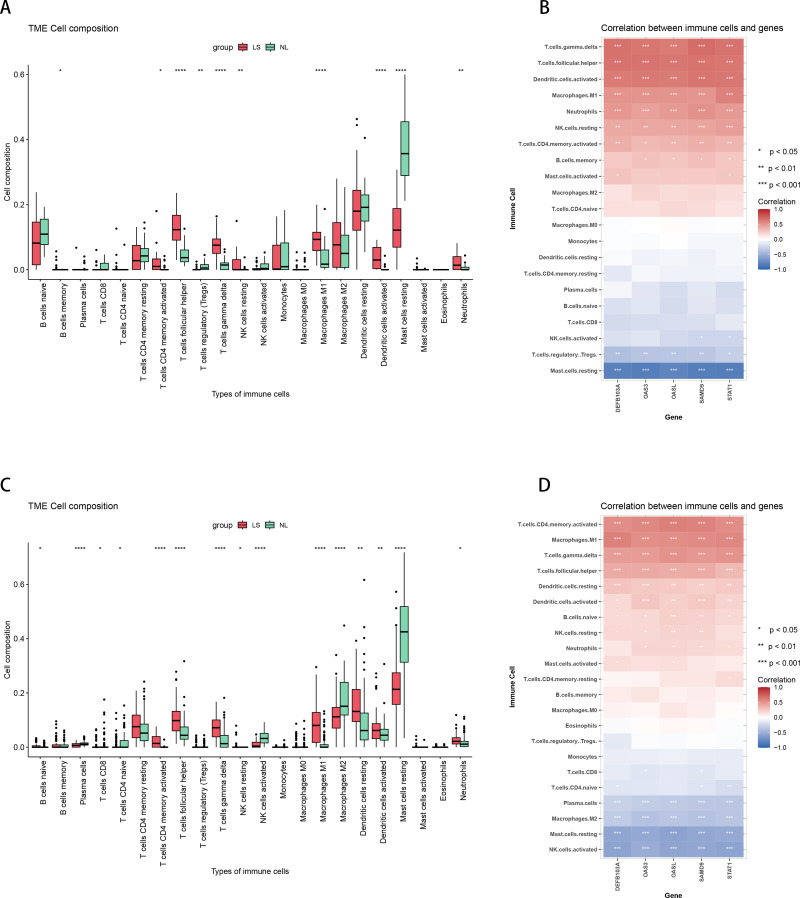
Immune cell infiltration. (A) The immune cell infiltration in NL/LS cohort; (B) Correlation between characteristic genes and immune cell infiltration features. (C) The immune cell infiltration in GSE30999, (D) Correlation between characteristic genes and immune cell infiltration features in GSE30999.

The immune cell infiltration analysis was performed on the turquoise module identified by WGCNA in NL/LS cohort with differentially expressed genes for evaluating the immune characteristics. The abundance of T cells follicular helper, T cell gamma delta, Macrophages M1 and Dendritic cells resting was higher in lesion than non-lesion, and abundance of Mast cells resting was relatively low (Fig 7A). The results reflected the relationship between immune cells and clinical manifestations in the process of psoriasis. The 5 characteristic genes (DEFB103A, OAS3, OASL, SAMD9, STAT1) all exhibited a high degree of correlation with the immunocyte infiltration characteristics mentioned above. There was a significant positive correlation with T cell gamma delta, Dendritic cells resting, T cells follicular helper, Macrophages M1, and a significant negative correlation with Mast cells resting (Fig 7B). The 5 characteristic genes also revealed a strong correlation and consistency with immune cell infiltration signatures from external data GSE30999 (Fig 7C, 7D). The results of immune cell infiltration further confirmed the key role of these characteristic genes in the progression of psoriasis.

### 3.6. Gene identification based on the LASSO model

LASSO model was established based on the hub genes of NL/LS and pre/post-treatment, respectively (S10 Fig). Expression profiles of 16 hub genes in NL/LS cohort were analyzed using LASSO regression analysis with a reference value of Lambda.min = 0.0003361. Among them, 14 genes (ABCG4, CD24, DEFB103A, EPHB2, FABP5, HPSE, IFI16, KYNU, OAS3, OASL, PLA2G4D, SERPINB13, STAT1, TMPRSS11D) were identified and used to construct gene features based on non-zero regression coefficients. A total of 5 genes (BUB1, HERC6, ISG15, MXD1, NCAPG) were identified in pre/post treatment (Lambda.min = 0.00879).

### 3.7. Evaluation of the accuracy of the LASSO model using ROC curves

**Fig 8:**
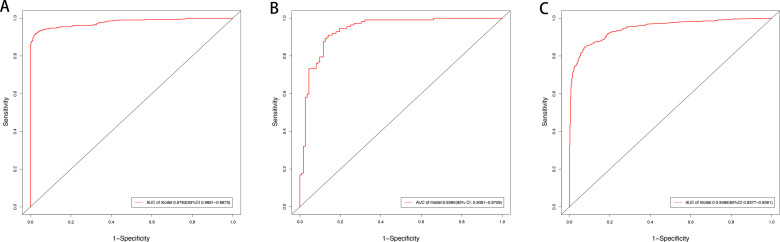
Establishment of a model for predicting psoriasis and its verification. (A) ROC curve analysis of NL/LS cohort; (B) ROC curve analysis of pre/post treatment cohort; (C) ROC curve analysis of external data GSE30999.

The AUC for the models of the NL/LS and pre/post treatment groups were 0.9873 (95% CI, 0.9691–0.9875) and 0.9395 (95% CI, 0.9081–0.9709), respectively (Fig 8A, B). Accuracy was further verified based on an external psoriasis dataset GSE30999, which contained expression profiles of 81 pairs of non-lesional/lesional tissues. Based on the hub genes identified gene above, AUC = 0.9469 (95% CI, 0.9377–0.9561) (Fig 8C). The ROC analysis demonstrated that this method effectively selected disease-associated feature genes. Therefore, the 14 up-regulated genes and 5 down-regulated genes identified based on LASSO model were closely related to the progression and treatment of psoriasis.

### 3.8. Analysis of short-term treatment gene sensitivity

The study drug in GSE 136757 was a TKY2/JAK1 inhibitor that specifically targets TYK2-dependent IL-12 and IL-23 signaling in T cells and keratinocytes, as well as downstream signaling pathways activated by JAK1-dependent signaling.

The correlation of 23 genes in pre/post treatment with the outcome of short-term clinical treatment (assessed by PASI scores, which evaluate the degree of improvement in psoriasis following 28 days of continuous medication) revealed that 8 genes (DEFB103A (P = 0.049), OASL (P = 0.026), HERC6 (P = 0.028), ISG15 (P = 0.014), MKI67 (P = 0.004), MX1 (P = 0.016), MXD1 (P = 0.029), SCO2 (P = 0.007)) had statistically significant differences in the prognostic sensitivity of the study drug. Thus, 8 biomarkers were considered to be highly sensitive in TYK2/JAK1, or highly correlated with the level of remission of psoriatic lesions.

## 4. Discussion

In this study, our main focus was on exploring of potential biomarkers associated with psoriasis and genes with high prognostic value related to clinical outcomes and treatment sensitivity. WGCNA was designed to identify co-expression modules and analyze them in modular units, linking genes, samples, modules, and traits. The scale-free weighted co-expression strategy clustering method of WGCNA was more consistent with biological phenomena, and presents the interaction relationship between genes, and it was easier to find the hub gene in regulatory center [28]. Considering that LASSO regression is efficient in processing high-dimensional data with more redundant features, this method was used to further analyze the highly correlated genes in the module [35]. In order to capture the co-expression relationship between genes comprehensively and retain meaningful biological information, we conducted a comprehensive analysis using original full transcriptional information and differential expressed genes. Therefore, based on WGCNA, the LASSO model and a series of validation methods, novel biomarkers (5 characteristic genes, 14 up-regulated genes, 5 down-regulated genes, and 8 prognostic sensitivity genes) were found to have clear biological significance.

DEFB103A, also known as HBD-3 (HDP human β-defensin-3), exhibited a significant upregulation in the expression levels within the lesional skin before treatment, followed by a significant downregulation of expression levels after treatment in this study [38–40]. In addition, there was a statistically significant difference in the short-term treatment sensitivity of psoriasis. Previous studies revealed that DEFB103A expression is significantly upregulated in patients with mild and moderate psoriasis vulgaris compared to healthy tissues [41]. Furthermore, previous studies have indicated that DEFB103A effectively stimulates the secretion of angiogenic growth factors, including fibroblast growth factor, platelet-derived growth factor, and vascular endothelial growth factor, subsequently inducing migration and proliferation of human fibroblasts. Meanwhile, DEFB103A enhances the phosphorylation of JAK2/STAT3, regulating the signal transduction of JAK-STAT in the IL-23/IL-17 axis that affects various cytokines (including IL-6, 12, 22, 23, 17, and IFN-γ) involved in intracellular signaling [38]. Therefore, JAK-STAT has become one of the key pathways in the treatment of psoriasis [16]. The data in this study came from clinical trials of inhibitors of the TYK2/JAK1, one of the JAK/STAT family pathways. These findings suggest that DEFB103A may be involved in the pathogenesis of psoriasis as a core gene, and participated in the treatment of psoriasis through the TYK2-JAK1 pathway.

IFI16 (Interferon-inducible protein 16) is an innate immune system sensor that can activate the function of keratinocytes, leading to the production of various immune-related proteins such as IL-1α, IL-1β, IL-8, CXCL1, CXCL10, CCL20, ICAM-1, and HLA-DR. This activation can lead to amplification of immune responses. In addition, IFI16 was highly expressed in the skin lesions that imiquimod-induced psoriasis mouse model. Meanwhile, the psoriasis lesions were improved when IFI16 expression was inhibited [42,43]. IFI16 was also one of 6 characteristic genes that were repeatedly identified in hub genes both in NL/LS and pre/post treatment. There are several prominent nodes (IL-1β, CXCL1, CCL20) in the network gene pathway diagram, which are related to the molecular mechanism disclosed by IFI16. It shows that IFI16 plays an important role in the pathogenesis and treatment of psoriasis.

OASL (Oligoadenylate synthetases-like) and OAS3 (2’-5’-oligoadenylate synthetase 3) are both members of the family of OASs (Oligoadenylate synthetases) [44]. As previously confirmed, the expression levels of OASL/OAS3 are extremely low in healthy skin. In patients with psoriasis, OAS3 regulates the cell cycle and enhances JAK1-STAT1 phosphorylation induced by type I interferon. This, in turn, promotes the proliferation of epidermal keratinocytes involved in the formation of the stratum corneum. Additionally, it has been observed that the expression level of OAS3 is positively correlated with the expression level of the cytokine IL-17A. It is speculated that viral infection can activate the JAK-STAT signaling pathway, promote cell proliferation by inducing OAS3 expression in keratinocytes, and ultimately aggravate the inflammatory response in psoriasis. OASL is induced by both type I and type II interferons, which are widely believed to be part of the antiviral mechanism. Numerous transcriptomic studies have shown that OASL is upregulated in response to various intracellular bacterial pathogen infections. The high expression of OASL suggests its association with RNA virus infection. In the response to RNA virus infection, OASL acts as an antiviral protein by enhancing signaling through the RIG-1 pathway and stimulating the production of IFNα and IFNβ [45]. This finding also confirms the enrichment of many virus-related pathways identified in our KEGG analysis. However, it can restrict the response of type I IFN during DNA virus infection [46]. Therefore, OASL may have dual functionality and demonstrate both inhibitory and promotive functions within cells. In another study based on the expression profile analysis of psoriasis vulgaris diffuse genes, OASL/OAS2/OAS3 were considered as novel hub genes associated with psoriasis. In addition, other hub genes included HERC6, ISG15, and MX1 [47].

STAT1 belongs to the family of the STAT, participates in the JAK/STAT signaling pathway in the expression of different transcription factors, as well as in the signaling cascade of type I and type II interferons. In recent decades, the JAK/STAT pathway has been suggested to play a key role in the pathogenesis of psoriasis. Relevant studies demonstrated a significant increase in the expression and activity of STAT1 in lesional psoriatic skin compared to non-lesional psoriatic skin. Luciferase assays have demonstrated that stimulation of cultured human keratinocytes with IFN-α or IFN-γ results in a prominent induction of STAT1 transcriptional activity [48]. Another study revealed that STAT1 inhibits the expression of IL-22 by directly antagonizing STAT3 and altering the balance between STAT1 and STAT3 activation, thereby inducing various severe skin inflammations [49,50]. STAT1 was also one of the 6 characteristic genes repeatedly identified. Therefore, its biological function in psoriasis needs to be further explored.

There is currently no direct evidence to support a direct association between ABCG4 and the progression of psoriasis. However, revealed by certain studies the fact that ABCG4 emerges as one of the most significantly up-regulated genes in warts, which are skin lesions caused by Human papillomavirus (HPV) infection. In the process of HPV-induced carcinogenesis, it is primarily HPV infection that stimulates the production of IL-17 throughout the body. HPV infection provides more favorable conditions for the secretion of IL-17. This mechanism aligns with the pathogenic mechanism of psoriasis and may potentially further facilitate the development of psoriasis [51–53].

CD24 and HPSE have been demonstrated with high expression in the upper layers and more differentiated layers of the psoriatic epidermis, contributing to alterations in the extracellular matrix [54,55]. LINC01215 has also been identified as one of the promising biomarkers for the diagnostic efficacy of psoriasis in a previous study [56].

FABP5, also known as epidermal or psoriasis-associated FABP, was initially identified in the psoriatic epidermis, hence its name [57]. Previous studies revealed that FABP5 is overexpressed in keratinocytes of psoriatic skin after mitosis. Inhibitors of FABP5 or downregulation of FABP5 expression can significantly improve inflammation and lipid metabolism in psoriasis. FABP-5 was positively correlated with inflammatory parameters such as CRP and white blood cell counts, which may indicate that it can be used as one of the indicators of inflammation in psoriasis patients [58,59].

In patients with chronic inflammatory skin disease, such as psoriasis and atopic dermatitis (AD), another tryptophan metabolism enzyme downstream of IDO (indoleamine 2,3-dioxygenase), KYNU is heavily upregulated [60]. The discernible upregulation of KYNU expression demonstrated a significant positive correlation with the manifestation of high disease severity and heightened inflammation in the context of psoriasis or AD [60–62]. Remarkably, after the implementation of effective treatments for these conditions, a noteworthy decrement in KYNU expression was observed. This suggests that down-regulated KYNU expression successfully reduced disease burden and improved inflammatory processes. However, the role of KYNU has not been explored in patients with these skin diseases or in general human immunology.

There is research showing a high expression of PLA2G4D in the cytoplasm of mast cells in psoriatic lesions. In a study by Cheung and colleagues, it was found that PLA2G4D is present on CD1a+ Langerhans cells, where it is recognized by lipid-specific T cells, leading to the production of abundant IL-22 and IL-17A [63]. Although this discovery of a self-antigen provides meaningful insights into the pathophysiology of psoriasis, its role in genetically susceptible individuals triggered by specific environmental factors is still not clear.

TMPRSS11D, commonly referred to as Human airway trypsin-like protease (HAT), is a significant physiological enzyme predominantly found in the respiratory tract. It plays a crucial role in promoting cell growth and facilitating the production of IL-8 [64,65]. The level of expression and the biological mechanisms underlying HAT in the skin remain poorly understood. Nevertheless, findings from RT-PCR studies have shown high expression of HAT in the epidermis of psoriasis patients, suggesting a potential role of TMPRSS11D in promoting the production of IL-8 mediated through protease-activated receptor 2 (PAR-2) [65]. In turn, it could contribute to the accumulation of inflammatory cells in the epidermal layer of psoriatic skin.

In a previous transcriptomic analysis of mild and severe plaque psoriasis, BUB1 has been identified as one of the central hub genes in severe psoriasis [66]. However, its biological significance needs further validation.

CCNB1 (Cyclin B1) has been considered to support the release of key molecular targets in psoriasis through the regulation of mast cell activation and macrophage polarization [67].

Regarded as an important antiviral protein is Interferon-stimulated gene 15 (ISG15). Previous studies revealed that the cell cycle encounters interruption during the G1/S transition phase if ISG15 was absent in a psoriasis cell model, thereby reducing the proliferation of keratinocyte cells responsible for epidermal formation [68]. Furthermore, decreased expression of the cell cycle-related protein cyclin D1 was also observed during this period. In addition, MX1 has been considered to be important antiviral proteins, which was found that up-regulated in psoriasis lesions in this study [69,70].

In a genetic study exploring small insertions and deletions, it is proposed that the NCAPG gene encodes a subunit of the condensin protein complex [71]. This subunit is responsible for chromosome condensation and stability during mitosis and meiosis. It is considered possible that NCAPG may function as a low-frequency frameshift gene and participate in the development of psoriasis.

Some biomarkers which are not well understood at present, were identified. There is no direct evidence that EPHB2 is involved in the pathogenesis of psoriasis, but previous studies have supported that EPHB2 is involved in Th17 cell differentiation and plays a key role in the antifungal signaling pathway. SAMD9 is an antiviral factor and tumor suppressor, which is a key factor in the development of congenital immune defense against poxvirus and myeloid tumors. SAMD9 is believed to have a NOD-like receptor structure, so it is consistent with the pathway enrichment results of KEGG, but the function and structure are unknown [72]. Previous studies found that MKI67 has biological functions in cell proliferation and cell cycle regulation, while more studies on SCO2 are believed to be related to mitochondrial defects [73]. MXD1 has been identified as a potential biomarker associated with specific molecular changes and tumor microenvironment characteristics in esophageal squamous cell carcinoma, but biological functions in psoriasis or immunology have been less reported [74,75].

Additionally, we have explored the biological processes and signaling pathways associated with the onset and treatment of psoriasis. GO enrichment analysis reveals that modules strongly associated with the etiology and clinical prognosis of psoriasis participate in biological processes such as immunity, cell cycle, and cytokine regulation. KEGG pathway enrichment result of the modules highly related to disease progression and treatment showed that Th17 (IL-17) as the core cytokine interaction and NOD-like receptor signaling pathway might be crucial pathways mechanism of psoriasis. In addition to repeated recognition of multiple signaling pathways, the high duplication enrichment with rheumatoid arthritis (RA) also suggests that psoriasis and RA may have a high degree of similarity in pathogenesis and treatment.

Moreover, the network diagram also reveals multiple core genes and transcripts directly related to cellular pathways associated with the onset and treatment of psoriasis, including IL-1B, IL-6, IL-17A, IL-18, CXCL8, INFG, STAT1, CD80, etc. These key nodes are highly correlated with the enriched pathway, suggesting that the biological mechanism of biomarkers such as interleukin and interferon may be one of the core mechanisms in the pathogenesis and treatment of psoriasis.

Multiple hub genes and signaling pathways associated with the progression and treatment of psoriasis were identified through WGCNA and LASSO regression analysis in full transcripts and differential gene transcripts. Most of the hub genes in the NL/LS cohort could be identified and validated by the hub gene sets of external validation datasets (GSE14905\GSE6710). Some of the hub genes in the pre/post-treatment (TYK2/JAK1) cohort were also identified by GSE14905 and GSE6710 at opposite expression levels as key genes, although GSE14905 and GSE6710 were not included in any pre/post-treatment psoriasis samples. Therefore, the biological functions of these hub genes may be highly consistent during the pathogenesis of psoriasis and TYK2/JAK1 treatment.

ROC curves verified that the hub genes with LASSO regression performed well in NL/LS cohort and in the pre/post-treatment cohorts for the specificity and sensitivity of genetic disease prediction. In addition, the results of immune cell infiltration in GSE136757 and validation dataset GSE30999 were consistent with the correlation between 5 characteristic genes and immune cells. However, there are several limitations that might be considered in this study, including the lack of in vivo and in vitro experimental validation, the classification of psoriasis severity, overfitting/underfitting in LASSO regression and the relevance of clinical treatment methods to psoriasis treatment pathways. Through the discovery of key genes that are highly related to the occurrence and prognosis of psoriasis, timely drug intervention can improve the therapeutic effect of psoriasis and reduce the life stress and disease burden of patients. In future studies, we will closely monitor relevant information from other databases and conduct in vitro research, with particular emphasis on the mechanisms underlying the role of the 5 characteristic genes (DEFB103A, OAS3, OASL, SAMD9, STAT1), 8 clinical outcome sensitive genes (DEFB103A, OASL, HERC6, ISG15, MKI67, MX1, MXD1, SCO2) and their associated pathways in psoriasis.

## 5. Conclusion

In summary, the study identified a series of characteristic genes (DEFB103A, IFI16, OAS3, OASL, SAMD9, STAT1, etc.) that are highly related to the occurrence, treatment of psoriasis. Besides, the functional enrichment of modules with high correlation with clinical traits and the correlation analysis of immune cell infiltration of characteristic genes, which provide a new perspective for further study of psoriasis.

## Supporting information

S1 Fig The heat map of differentially expressed genes. (A) The heat map of the 50 most differentially expressed genes in NL/LS cohort. (B) The heat map of the 50 most differentially expressed genes in pre/post treatment cohort.
(PDF)

S2 Fig Cluster outlier detection of NL/LS cohort. (PDF)

S3 Fig Cluster outlier detection of pre/post treatment cohort. (PDF)

S4 Fig Chord diagram: Go analysis of the key module in NL/LS cohort (Biological process). (PDF)

S5 Fig Chord diagram: Go analysis of the key module in NL/LS cohort (Cellular component). (PDF)

S6 Fig Chord diagram: Go analysis of the key module in NL/LS cohort (Molecular function). (PDF)

S7 Fig Chord diagram: Go analysis of the key module in pre/post treatment cohort (Biological process). (PDF)

S8 Fig Chord diagram: Go analysis of the key module in pre/post treatment cohort (Cellular component). (PDF)

S9 Fig Chord diagram: Go analysis of the key module in pre/post treatment cohort (Molecular function). (PDF)

S10 Fig The results of LASSO model. (A) LASSO model of NL/LS cohort; (B) LASSO model of pre/post treatment cohort.
(PDF)

S1 Table Differentially expressed genes in the NL/LS cohort. (XLS)

S2 Table Differentially expressed genes in the pre/post treatment cohort. (XLS)

S3 Table The results of hub genes in GSE14905 and GSE6710. (XLS)

S4 Table The results of GO enrichment analysis in NL/LS cohort. (XLS)

S5 Table The results of KEGG enrichment analysis in NL/LS cohort. (XLS)

S6 Table The results of GO enrichment analysis in pre/post treatment cohort. (XLS)

S7 Table The results of KEGG enrichment analysis in pre/post treatment cohort. (XLS)

## Abbreviations

WGCNA: Weighted Gene Co-expression Network Analysis
LASSO: Least Absolute Shrinkage and Selection Operator
GSEA: Gene set enrichment analysis
GO: Gene Ontology
KEGG: Kyoto Encyclopedia of Genes and Genomes
DEGs: Differentially expressed genes
ROC: Receiver operating characteristic
NL: Non – lesional
LS: Lesional
AUC: Area under curve
TYK: Tyrosine Kinase
JAK: Janus kinase
STAT: Signal transducer and activation of transcription
GEO: Gene Expression Omnibus
BP: Biological process
CC: Cellular component
MF: Molecular function
TOM: Topological overlap matrix
MEs: Module eigengenes
GS: Gene significance
MM: Module membership
IL: Interleukin
IFN-γ: Interferon-γ
TNFα: Tumor necrosis factor
HPV: Human papillomavirus
AD: Atopic dermatitis
RA: Rheumatoid arthritis.
