# Supplementary figures and images for: Identification of novel biomarkers related to pathogenesis and treatment of psoriasis based on integrated analysis of weighted gene co-expression network analysis and LASSO
Source: PLoS One. 2025 Jun 25;20(6):e0317666. doi: 10.1371/journal.pone.0317666 (PMC12192183; doi:10.1371/journal.pone.0317666)

A

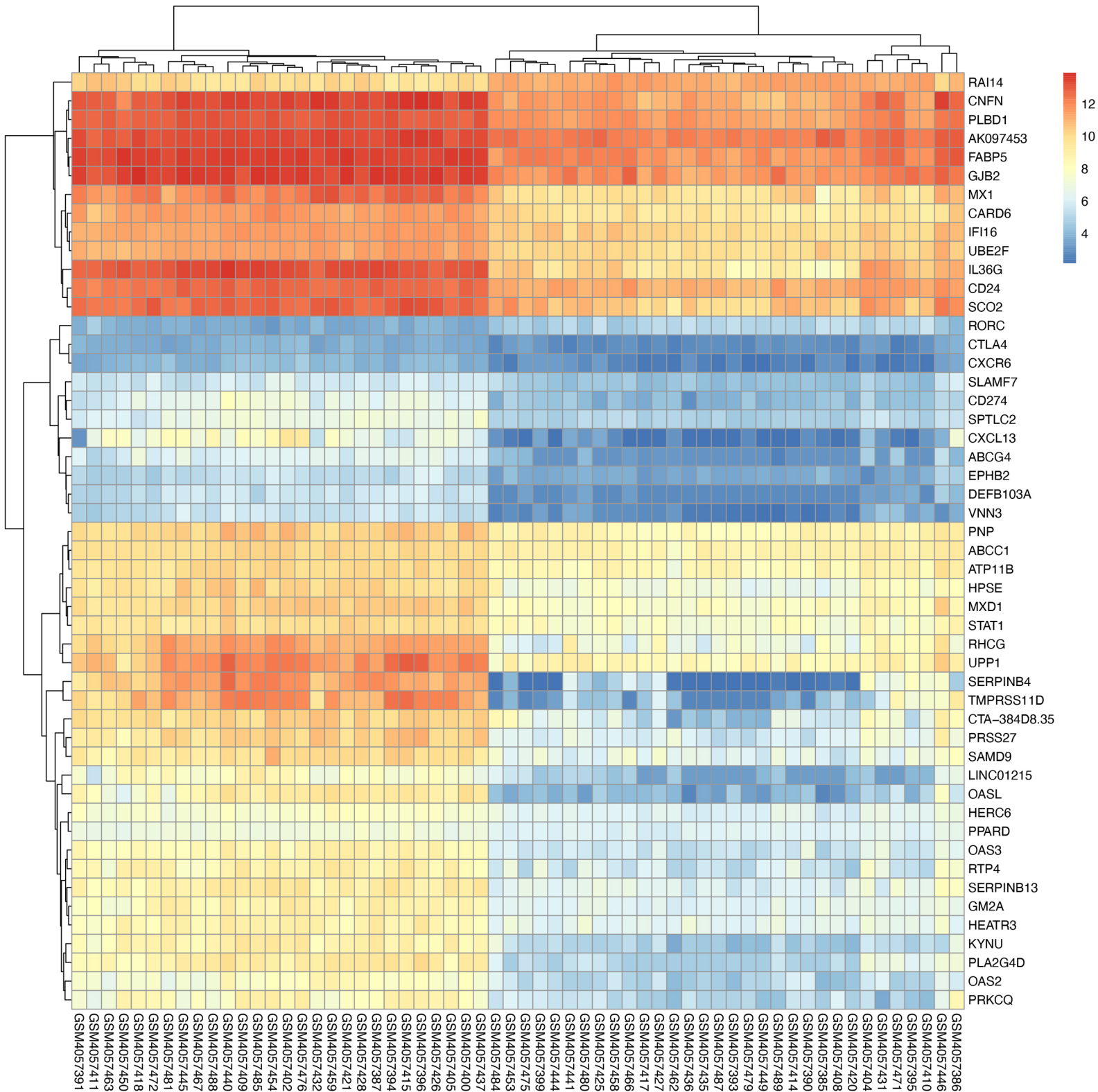

B

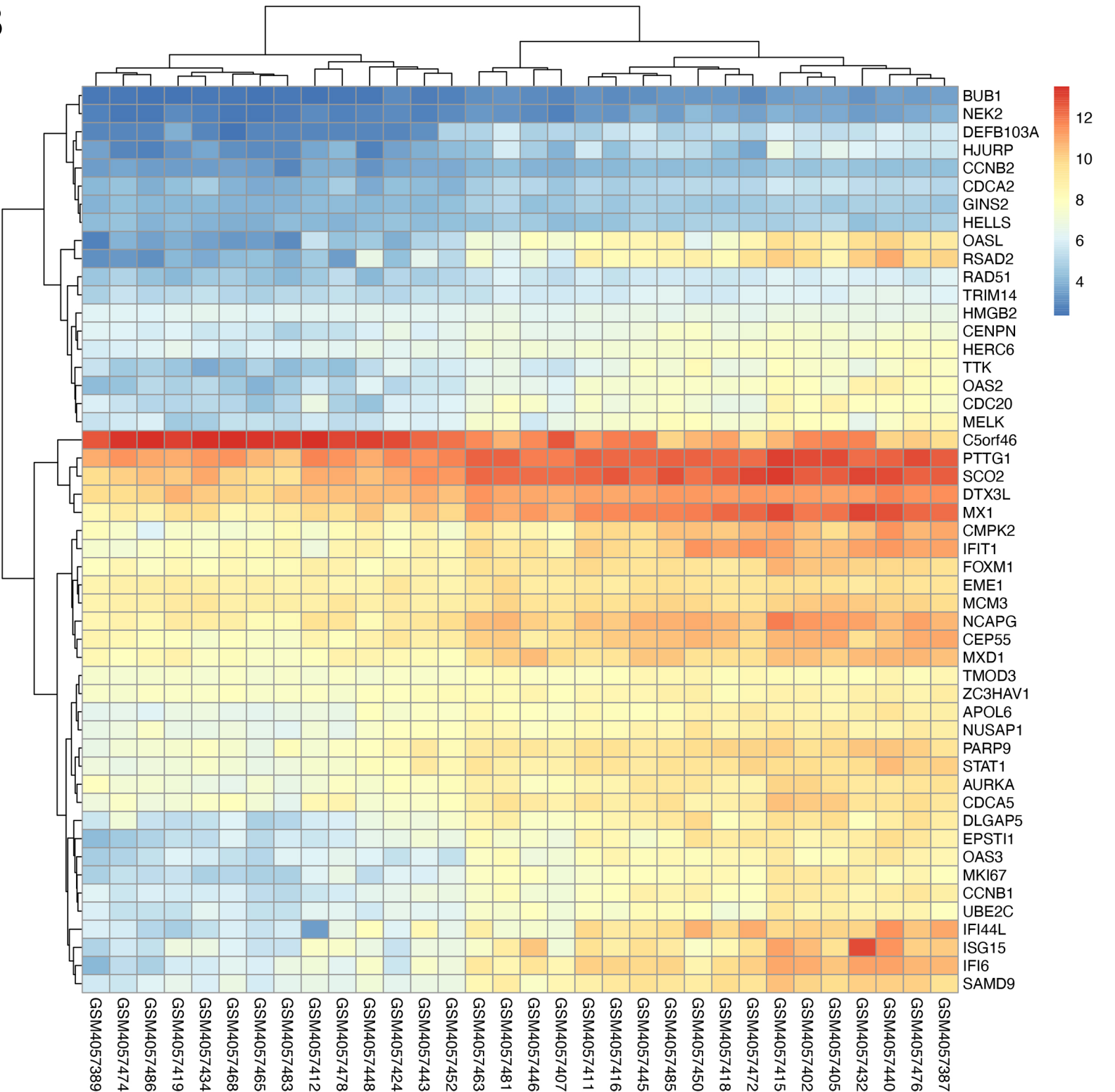

Supplement: S1 Fig — (A) The heat map of the 50 most differentially expressed genes in NL/LS cohort. (B) The heat map of the 50 most differentially expressed genes in pre/post treatment cohort. (PDF) [file pone.0317666.s001.pdf]

Sample clustering to detect outliers

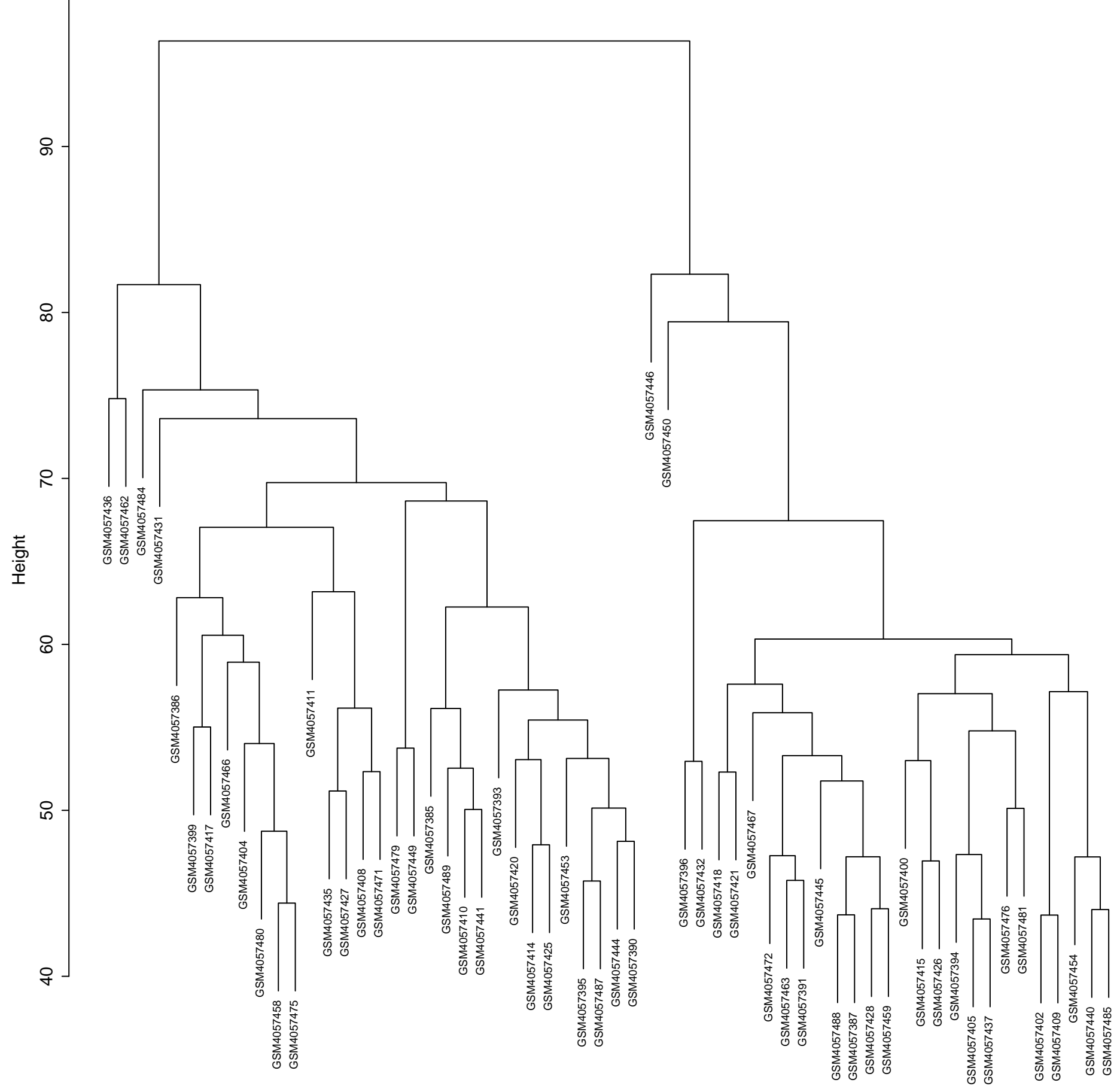

Supplement: S2 Fig — (PDF) [file pone.0317666.s002.pdf]

Sample clustering to detect outliers

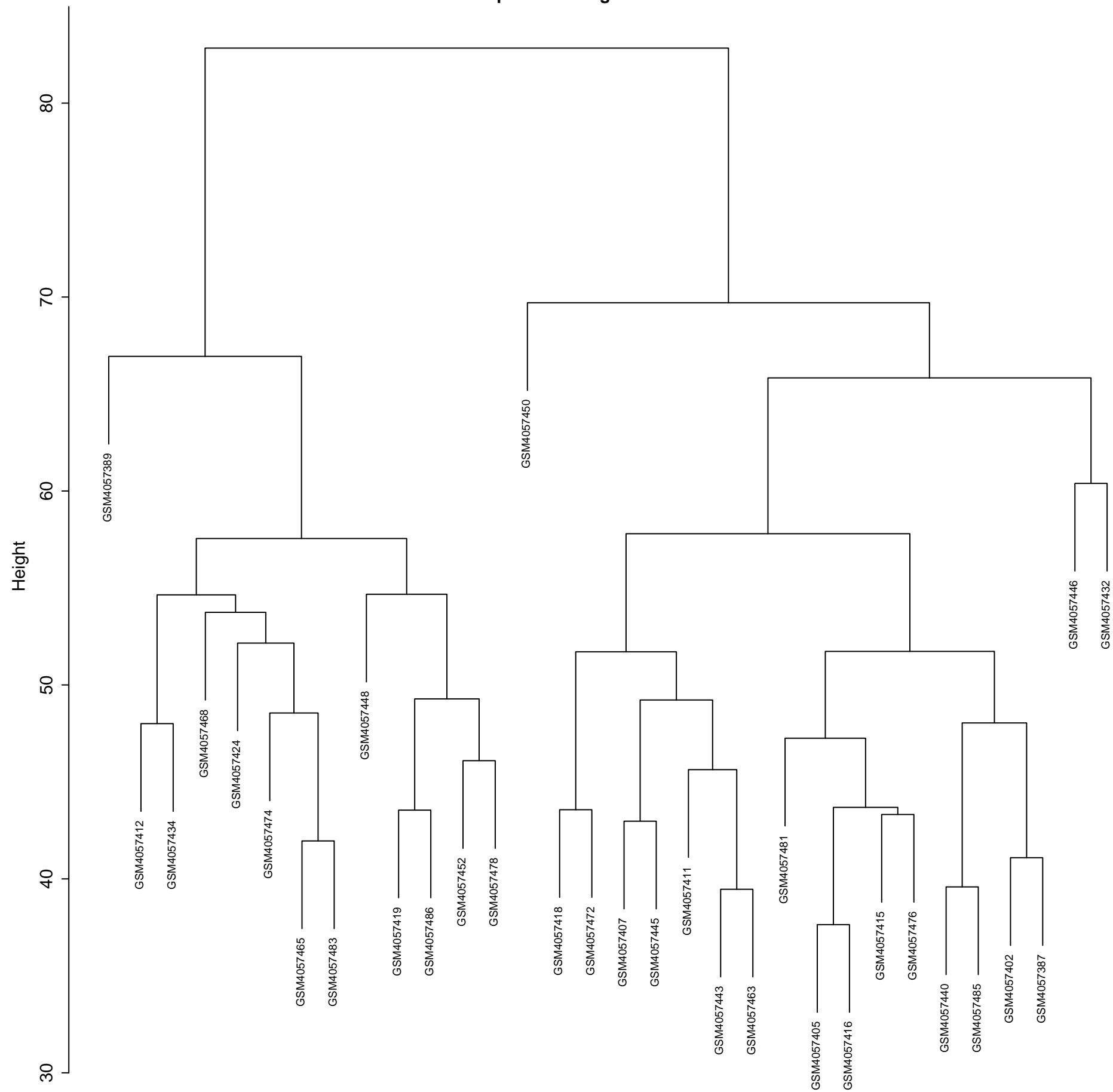

Supplement: S3 Fig — (PDF) [file pone.0317666.s003.pdf]

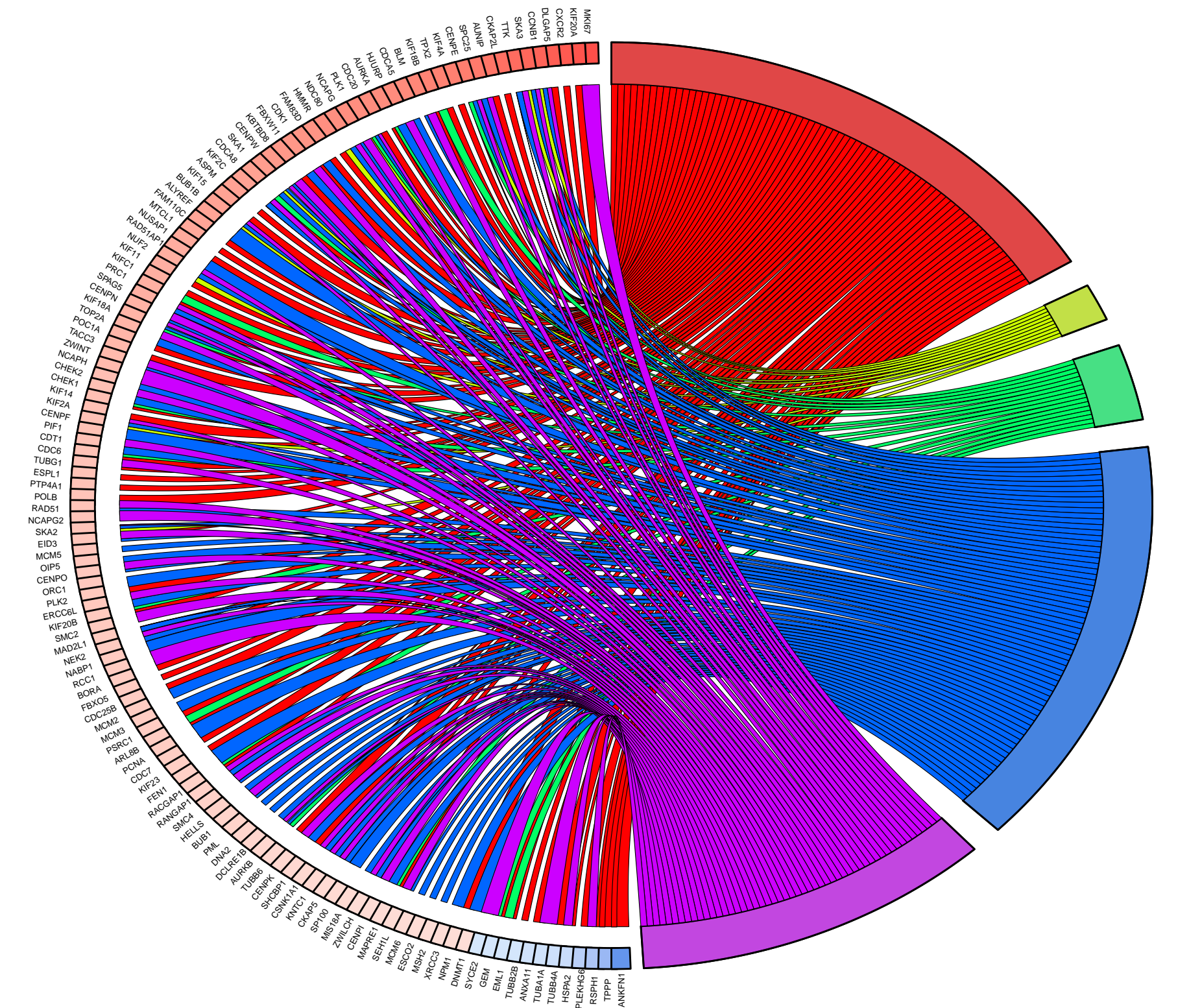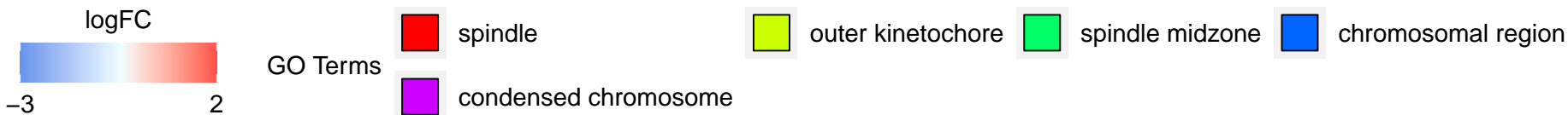

Supplement: S5 Fig — (PDF) [file pone.0317666.s005.pdf]

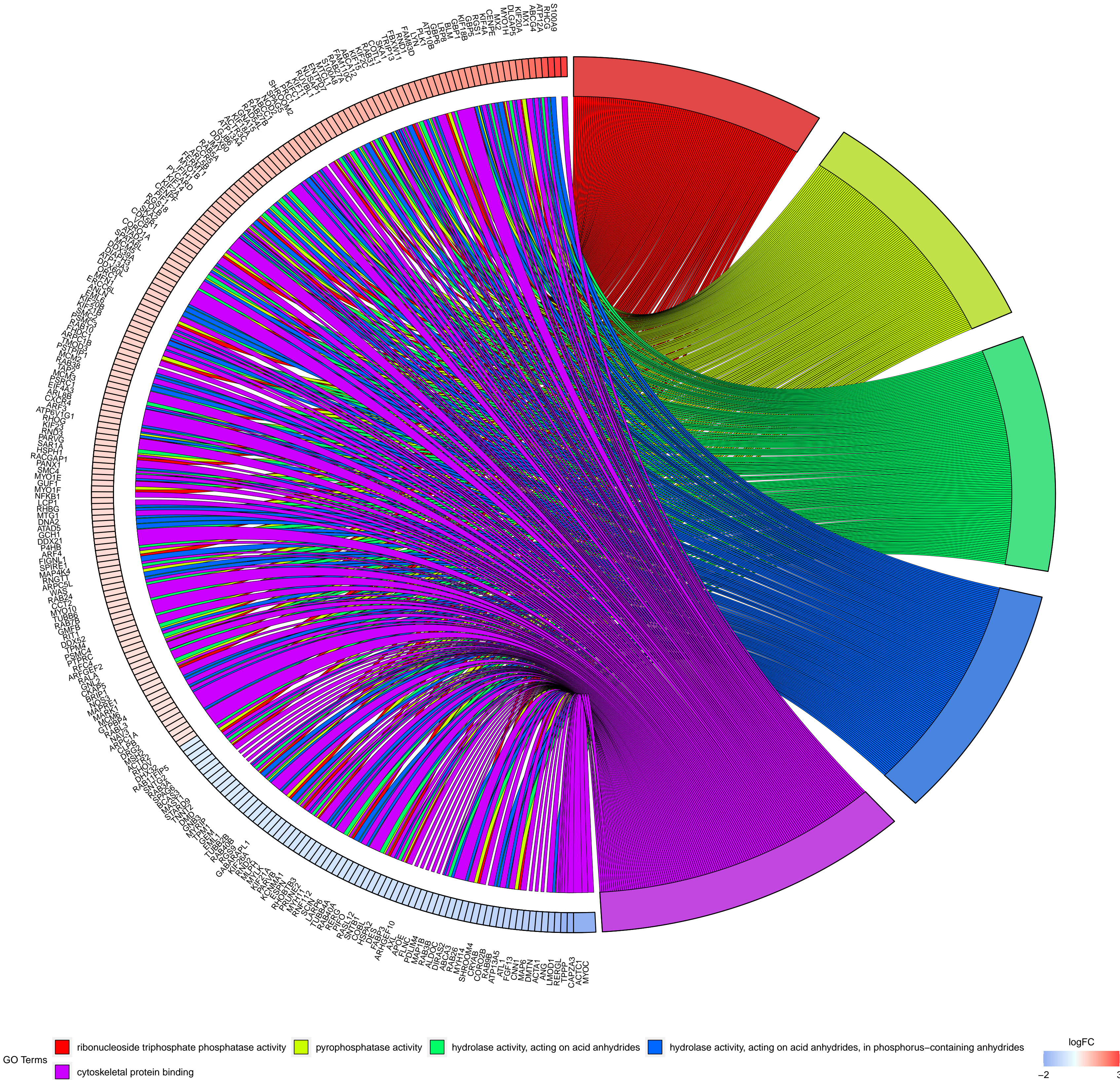

Supplement: S6 Fig — (PDF) [file pone.0317666.s006.pdf]

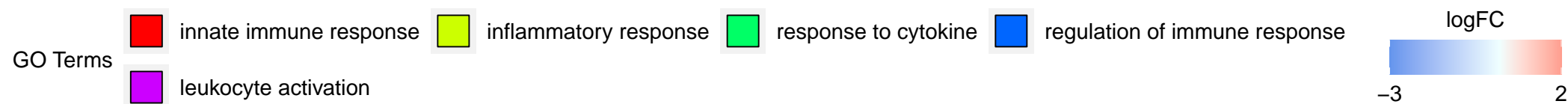

Supplement: S7 Fig — (PDF) [file pone.0317666.s007.pdf]

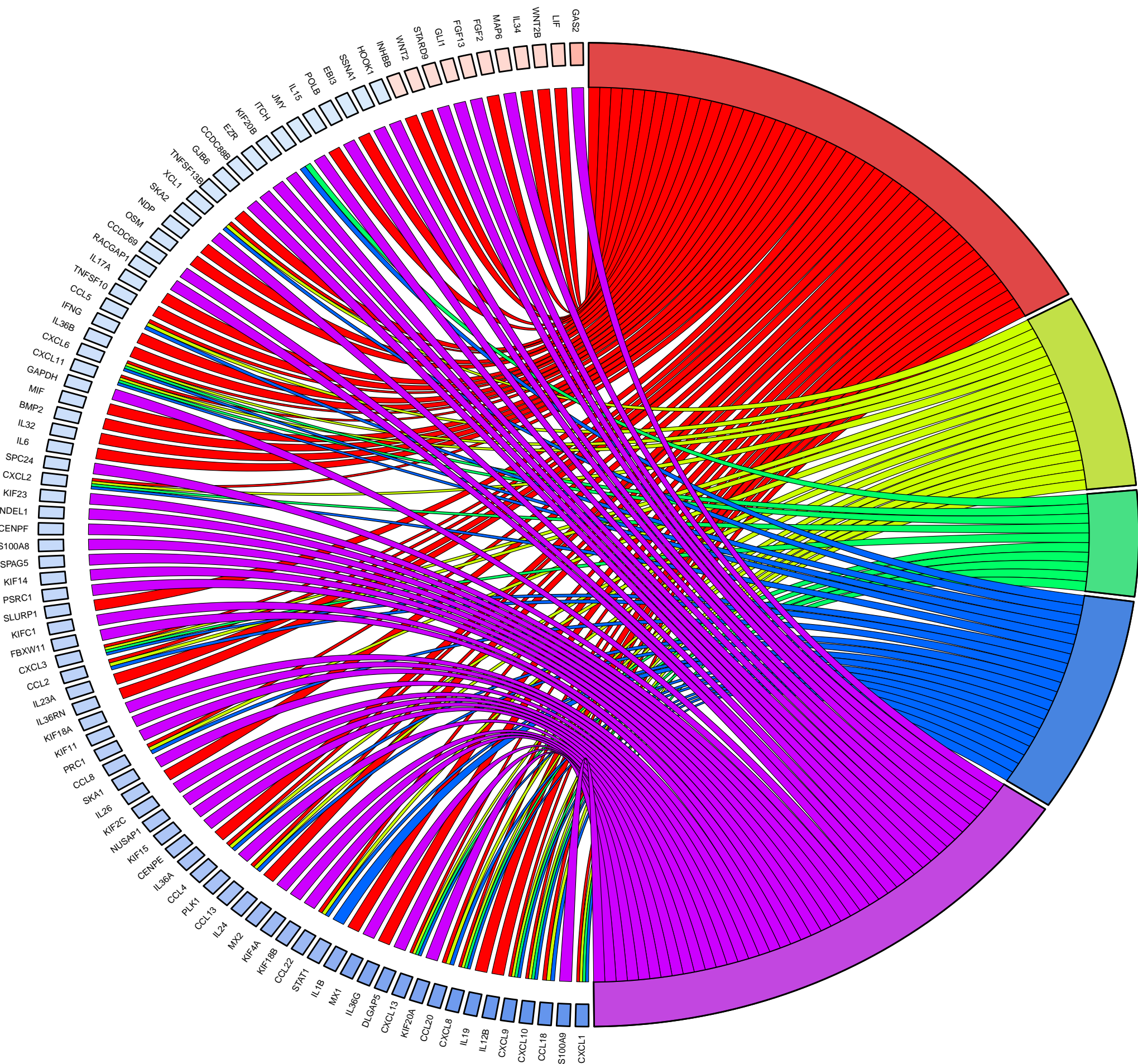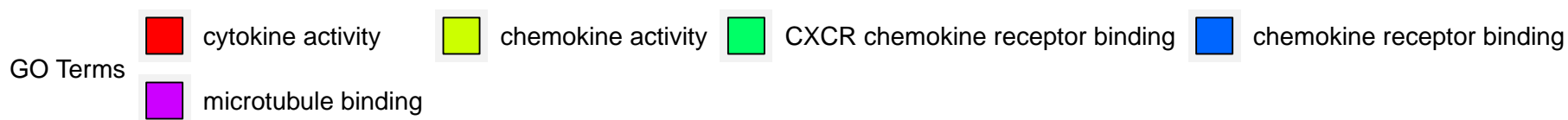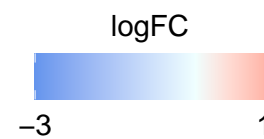

Supplement: S9 Fig — (PDF) [file pone.0317666.s009.pdf]

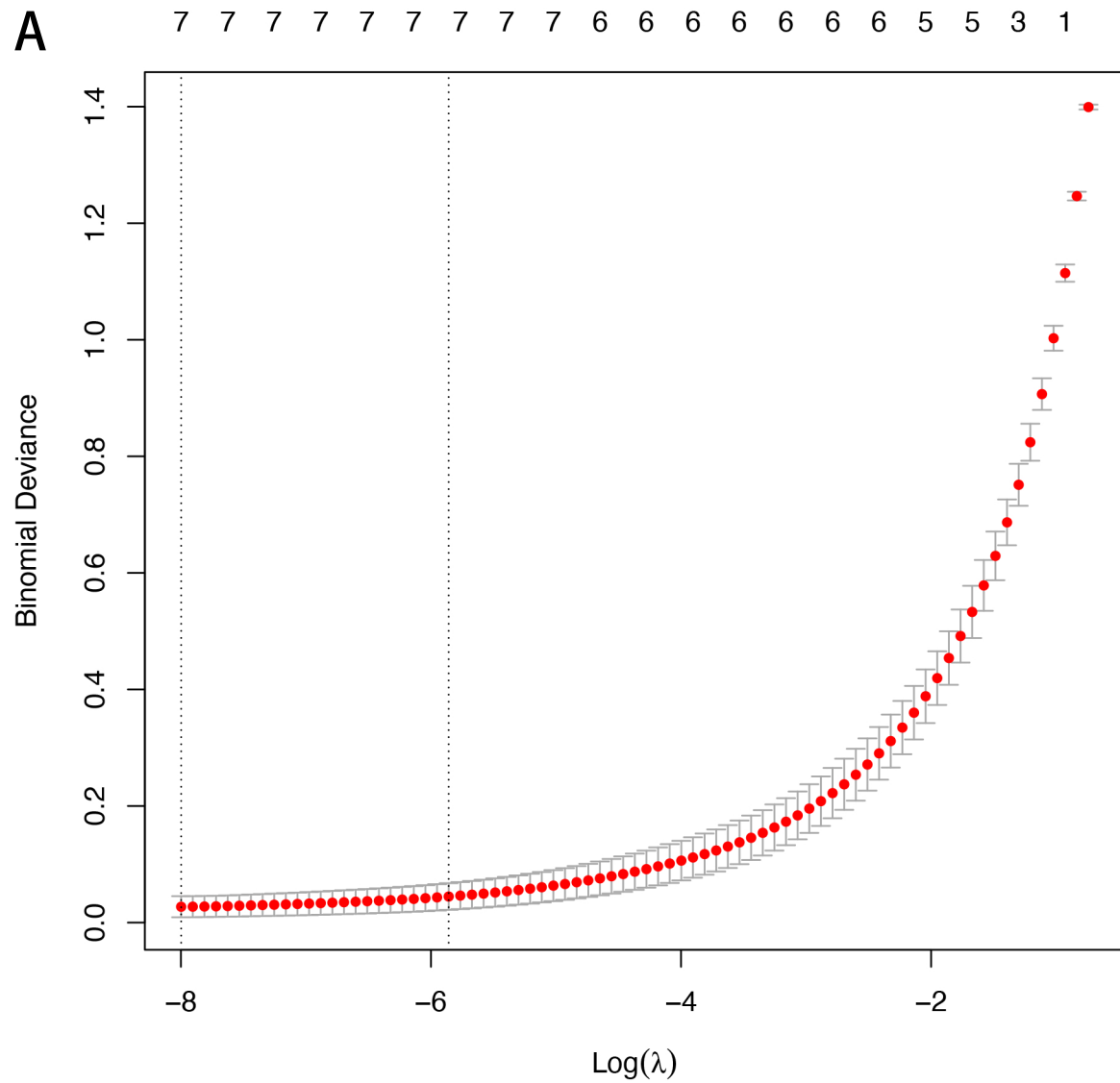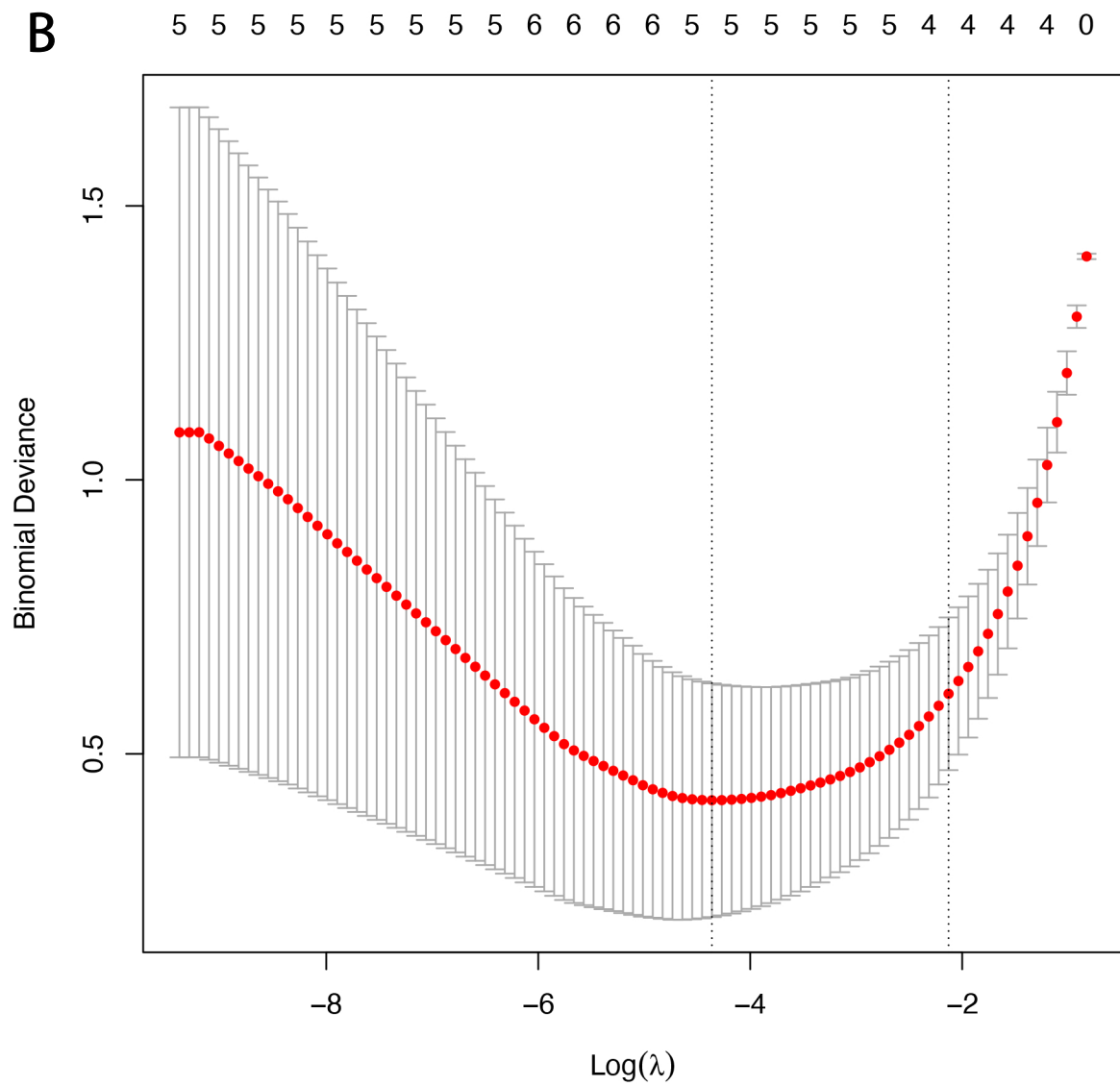

Supplement: S10 Fig — (A) LASSO model of NL/LS cohort; (B) LASSO model of pre/post treatment cohort. (PDF) [file pone.0317666.s010.pdf]
