# Supplementary material for: Identification of novel biomarkers related to pathogenesis and treatment of psoriasis based on integrated analysis of weighted gene co-expression network analysis and LASSO
Source: PLoS One. 2025 Jun 25;20(6):e0317666. doi: 10.1371/journal.pone.0317666 (PMC12192183; doi:10.1371/journal.pone.0317666)
Supplement: S4 Fig — (PDF) [file pone.0317666.s004.pdf]

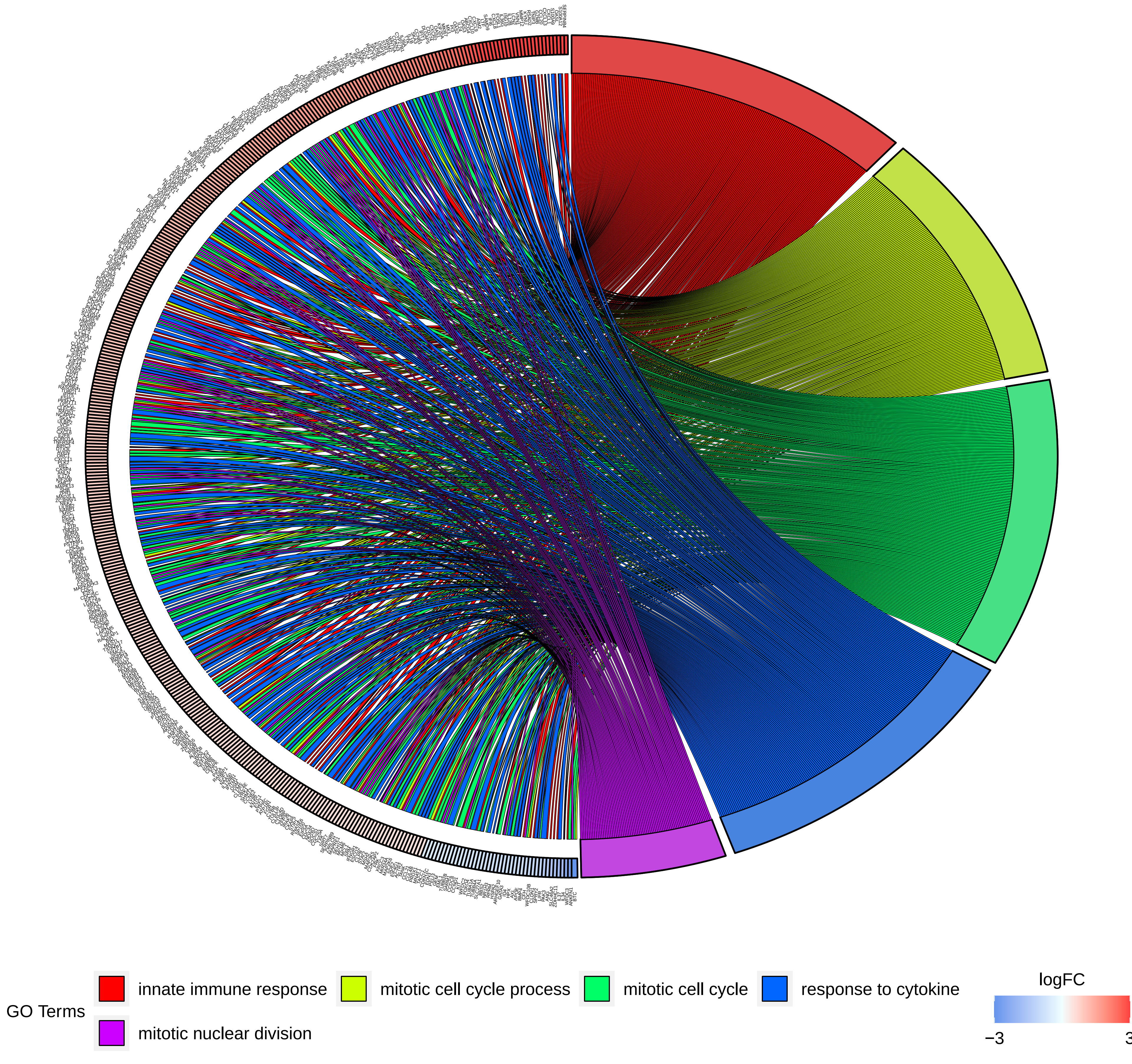
GO Terms
innate immune response
mitotic cell cycle process
mitotic cell cycle
response to cytokine
mitotic nuclear division
logFC
−3
3
