# Supplementary material for: Identification of novel biomarkers related to pathogenesis and treatment of psoriasis based on integrated analysis of weighted gene co-expression network analysis and LASSO
Source: PLoS One. 2025 Jun 25;20(6):e0317666. doi: 10.1371/journal.pone.0317666 (PMC12192183; doi:10.1371/journal.pone.0317666)
Supplement: S8 Fig — (PDF) [file pone.0317666.s008.pdf]

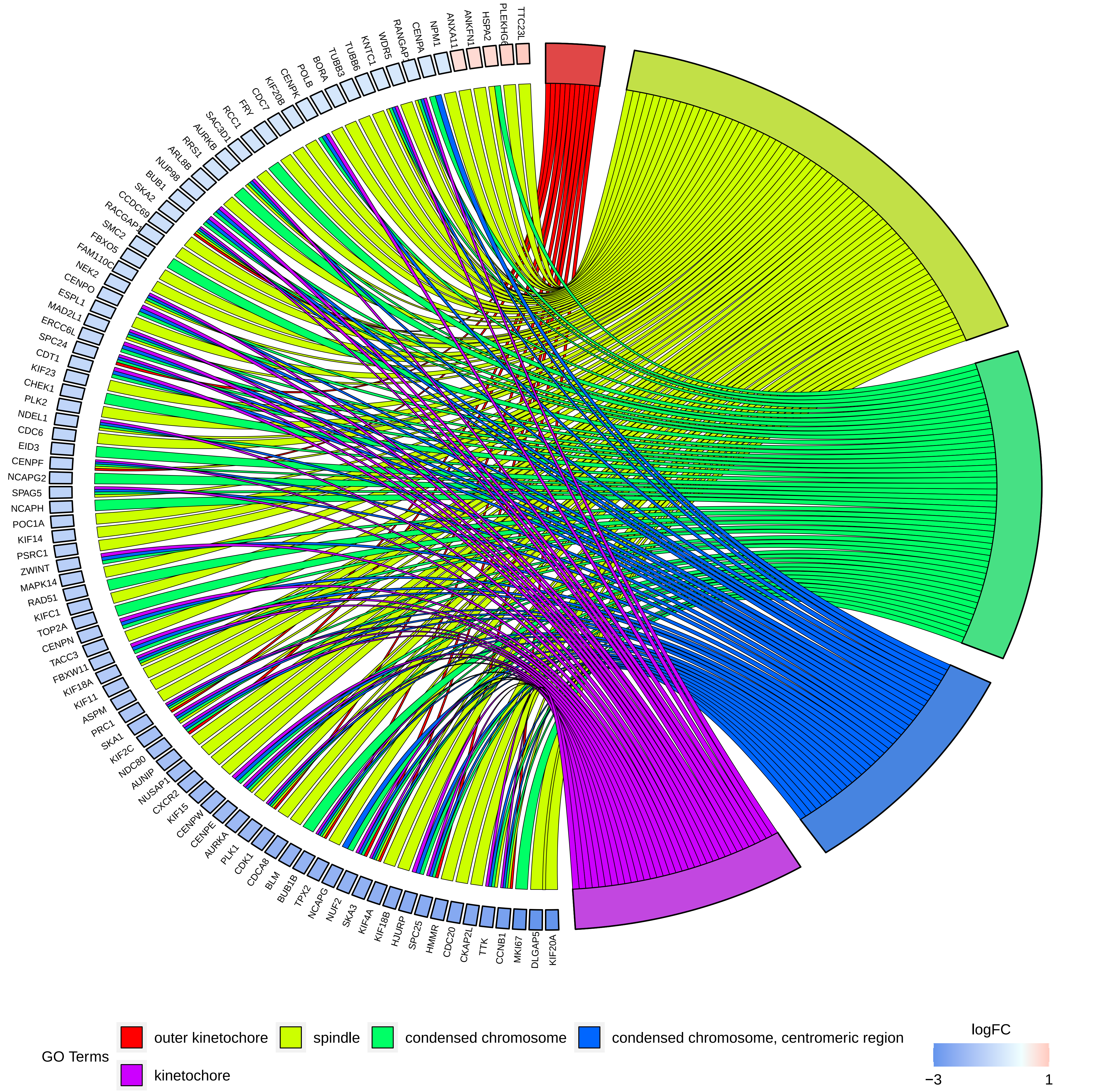
TTC23L
PLEKHG6
HSPA2
ANKFN1
ANXA11
NPM1
CENPA
RANGAP1
WDR5
KNTC1
TUBB6
TUBB3
BORA
POLB
CENPK
KIF20B
CDC7
FRY
RCC1
SAC3D1
AURKB
RRS1
ARL8B
NUP98
BUB1
SKA2
CCDC69
RACGAP1
SMC2
FBXO5
FAM110C
NEK2
CENPO
ESPL1
MAD2L1
ERCC6L
SPC24
CDT1
KIF23
CHEK1
PLK2
NDEL1
CDC6
EID3
CENPF
NCAPG2
SPAG5
NCAPH
POC1A
KIF14
PSRC1
ZWINT
MAPK14
RAD51
KIFC1
TOP2A
CENPN
TACC3
FBXW11
KIF18A
KIF11
ASPM
PRC1
SKA1
KIF2C
NDC80
AUNIP
NUSAP1
CXCR2
KIF15
CENPW
CENPE
AURKA
PLK1
CDK1
CDCA8
BLM
BUB1B
TPX2
NCAPG
NUF2
SKA3
KIF4A
KIF18B
HJURP
SPC25
HMMR
CDC20
CKAP2L
TTK
CCNB1
MKI67
DLGAP5
KIF20A
GO Terms
outer kinetochore
spindle
condensed chromosome
condensed chromosome, centromeric region
kinetochore
logFC
−3
1
